# Hydrothermal Synthesis of Cellulose-Derived Carbon Nanospheres from Corn Straw as Anode Materials for Lithium ion Batteries

**DOI:** 10.3390/nano9010093

**Published:** 2019-01-12

**Authors:** Kaifeng Yu, Jingjing Wang, Kexian Song, Xiaofeng Wang, Ce Liang, Yanli Dou

**Affiliations:** 1 Key Laboratory of automobile Materials, Ministry of Education, and College of Materials Science and Engineering, Jilin University, Changchun 130025, China; yukf@jlu.edu.cn (K.Y.); jingjingw17@mails.jlu.edu.cn (J.W.); skxjlu@126.com (K.S.); 2 State Key Laboratory of Inorganic Synthesis and Preparative Chemistry, College of Chemistry, Jilin University, Changchun 130025, China; wangxf103@jlu.edu.cn

**Keywords:** biomass, corn straw cellulose, hydrothermal carbonization, carbon nanospheres, lithium ion battery

## Abstract

As a most attractive renewable resource, biomass has the advantages of low pollution, wide distribution and abundant resources, promoting its applications in lithium ion batteries (LIBs). Herein, cellulose-derived carbon nanospheres (CCS) were successfully synthesized by hydrothermal carbonization (HTC) from corn straw for use as an anode in LIBs. The uniform distribution and cross-linked structure of carbon nanospheres were obtained by carefully controlling reaction time, which could not only decrease the transport pathway of lithium ions, but also reduce the structural damage caused by the intercalation of lithium ions. Especially, obtained after hydrothermal carbonization for 36 h, those typical characteristics make it deliver excellent cycling stability as well as the notable specific capacity of 577 mA h g^−1^ after 100 cycles at 0.2C. Hence, this efficient and environment-friendly method for the fabrication of CCS from corn straw could realize the secondary utilization of biomass waste, as well as serve as a new choice for LIBs anode materials.

## 1. Introduction

With the shortage of fossil fuels and the emergence of environmental problems, there is a booming demand for clean energy to meet current and future energy needs [1,2,3,4,5]. There are many kinds of biomass resources, among which cellulose, hemicellulose and lignin are the main forms. Biomass is an economical and promising carbon material for the anode of lithium ion batteries (LIBs), which has attracted the attention of many experts and scholars [2,3,5]. At present, it is crucial to realize the comprehensive utilization of biomass for the development of the LIBs system [5,6,7,8,9]. In recent years, with the rapid development of new energy technology, it is a trend to prepare carbonaceous LIBs anode materials using biomass as raw materials [9,10,11,12,13]. As a kind of farmland waste, corn straw is a kind of non-pollution renewable resource. Using corn straw-derived carbon material as an electrode not only realizes the re-using of agricultural waste, but also provides a novel environment-friendly way to obtain negative carbon material for LIBs.

In order to enhance the energy density, cycle performance and rate performance of LIBs, many efforts have been made on the structure of anode carbon materials, such as carbon nanotube, carbon nanowire, carbon nanosphere, carbon nanorod and carbon nanocapsule [14,15]. Carbon nanosphere is an important structure of negative carbon materials, which is considered as a promising precursor of carbon electrode materials owing to the higher bulk density and higher volume specific capacity than natural graphite and other flake materials. Moreover, the spherical structure exhibits excellent thermal and chemical stability of insertion and extraction of lithium ions [16,17,18,19,20].

At present, hydrothermal carbonization is the main method to prepare carbon nanospheres, as well as an effective way to realize the thermochemical conversion of biomass. HTC has the advantages of a simple operation process, mild synthesis conditions and renewable raw materials, providing a broad prospect for the large-scale production of carbon nanospheres [21,22,23,24]. Zhao et al. prepared well-dispersed micrometer-sized carbon nanospheres from cellulose by citric acid catalysed HTC (200–240 °C), and the diameter of carbon nanospheres became wider with the increase of citric acid concentration, reaction temperature and reaction time [25]. Hao et al. reported a new technique for preparing porous carbon nanospheres by the HTC of waste sugar solution followed through KOH activation. The specific capacity of the carbon nanospheres delivered good cycle life (296.1 F g^−1^ at a current density of 40 mA g^−1^) and superior rate capability (1.5 A g^−1^ after 5000 cycles) [26]. Inada et al. prepared carbon nanospheres by HTC from glucose at 800 °C; the obtained carbon nanospheres possessed a highly specific surface area, low resistance and high capacitance (higher than commercial activated carbon electrode) [27]. Remarkably, the work published by Xie and coworkers reported the preparation of homogeneous nanospheres (120, 360 nm) by introducing a small amount of poly (4-styrenesulfonic acid-co-maleic acid) sodium salt (PSSMA) into the hydrothermal system. Nanospheres with an adjustable size in the range of 1.4 to 5.2 μm were prepared in the presence of PSSMA and hydrochloric acid and showed good performance in catalytic hydrogenation [28].

Up to the present, although great progress has been made in the preparation of carbon nanospheres, the application of carbon nanospheres in LIBs is still a great challenge. Complex preparation processes and low yield have become significant drawback for LIBs anodes, which hinder the commercial development of carbon nanospheres [6,13,29]. To address these issues, it is highly desirable to develop an economical and efficient method to prepare carbon nanospheres, which will circumvent these limitations, especially for large batteries. We propose, herein, an environmental and simple route to obtain cellulose-derived carbon nanospheres by using corn straw as raw material. Through controlling the reaction time, the cellulose-derived carbon nanospheres after HTC for 36 h display an excellent electrical conductivity and rate performance, opening up a new opportunity for LIBs anodes.

## 2. Materials and Methods

### 2.1. Materials Synthesis

Corn straw was collected from a farmland in Jilin Province, China. Corn straw was washed with deionized water and dried in oven at 60 °C for 24 h. The dried corn straw was pulverized into powder by a pulverizing machine. Thirty grams of corn straw power was added to 4% H_2_SO_4_ at 1:10 solid-liquid ratio at 90 °C water bath. The xylose acid solution and filtrate residue were obtained by separating the solution from the filter residue after the reaction. Filter residue I was washed to neutral and dried with deionized water. Then Lignin was removed from filter residue I and 50 g L^−1^ NaOH at 99 °C water bath at 1:15 solid-liquid ratio. Lignin lye solution and filter residue Ⅱ were obtained by separating the reaction solution from the filter residue. The filter residue Ⅱ was washed to neutral and dry with deionized water. After filtering, the solid was transferred to a stainless steel autoclave with water treated at 200 °C. The reaction time was carried out for 24, 36, 48 and 60 h, respectively. The powder was carbonized in a tubular furnace at 600 °C under argon atmosphere at a heating rate of 5 °C min^−1^. The obtained samples were washed with deionized water and ethanol to remove tar and other organics, and then were dried in an oven at 60 °C. The samples were labeled as CCS-24, CCS-36, CCS-48, CCS-60, respectively.

### 2.2. Material Characterizations

X-ray diffraction patterns of samples were measured with X-ray diractometer (Siemens D5000, Munich, Germany) with nickel-filtered Cu K radiation (λ = 0.15406 nm) at 4° min^−1^. Raman spectra was tested on a Renishaw inVia instrument (wavenumber range: 200–2000 cm^−1^, λ = 514 nm). Thermogravimetric analysis (TGA) of corn straw was accomplished on Q500 thermogravimetric analyzer (TA Instruments, New Castle, PA, USA) at a scanning rate of 5 °C min^−1^ from 20 °C to 800 °C under N_2_ atmosphere. The specific surface area and pore diameter were carried out using nitrogen adsorption–desorption measurements (Micromeritics, ASAP2420, Micromeritics Instrument, Norcross, GA, USA). The morphology of samples was characterized by scanning electron microscopy (SEM, JEOL-JSM-6700F, JEOL Ltd, Tokyo, Japan) and transmission electron microscopy (TEM, JEM-2100F, JEOL Ltd, Tokyo, Japan). The superficial area and pore size distribution of the carbons were observed through Micromeritics ASAP2420.

### 2.3. Electrochemical Measurement

The slurry was prepared by mixing CCS, acetylene black and PVDF with a mass ratio of 8:1:1 in a mixing bottle. After grinding evenly, N-methyl-2-pyrrolidone was added into the mixture and then magnetically stirred for 6 h. The agitated paste was smeared on the copper foil, followed by drying in a vacuum oven at 120 °C for 12 h. The dried anode plate was punched on the sheet press to form a circular plate with a diameter of 10 mm; the circular electrode plates were weighed and numbered. The electrode loading mass was 0.13 mg cm^−2^. Coin-type (CR2025) cells were assembled in a glove box filled with argon. The opposite electrode and reference electrode used a lithium metal sheet, and polypropylene microporous membrane was used as the diaphragm. EC and DMC were used as the electrolyte solvent with a volume ratio of 1:1; LiPF_6_ was used as electrolyte solute at 1 mol L^−1^. The electrochemical performance was tested after 12 h of battery assembly. Cyclic voltammograms and impedance curve were performed on an electrochemical workstation (CHI660C) within a voltage window of 0–3.0 V at a scan rate of 0.1 mV s^−1^. The charge–discharge performance was tested between 0.02 V and 3.0 V at a 0.2C (1C = 372 mA g^−1^) on a LAND (CT2001A) battery system.

## 3. Results

### 3.1. Preparation of CCS

**Figure 1:**
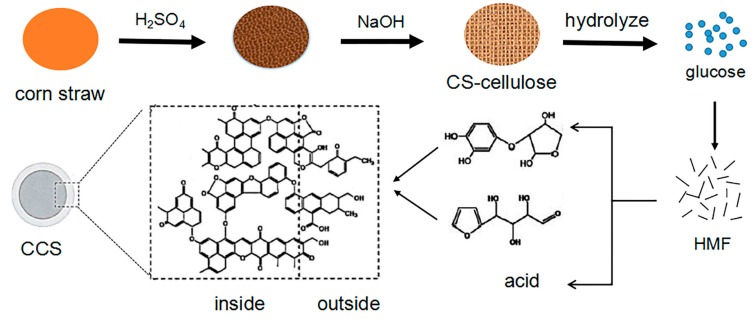
Schematic illustration of the CCS preparation.

Up to now, Lamer’s “nucleation-diffusion control” model has been widely used to explain the formation of carbon nanospheres in the hydrothermal process, including nucleation and growth [30,31]. At first, the aromatic nuclei are formed in the solution. When the concentration reaches the saturation critical value, the nucleation is produced, and then the crystal nuclei will grow under the joint action of diffusion and adsorption [29,30,31,32]. During the preparation of CCS, the morphology of carbon materials changed greatly from corn straw to carbon nanospheres. Figure 1 shows the schematic illustration of the formation of CCS. The corn straw was hydrolyzed under the action of acid to remove hemicelluloses and then boiled with alkali to remove lignin to obtain cellulose. After acidizing hydrolysis and alkali boiling of corn straw, cellulose with a block structure was obtained. The subsequent hydrothermal carbonation stage is the key to the formation of carbon nanospheres. Firstly, at a high temperature and high pressure, cellulose molecules hydrolyze in aqueous solution to form glucose. Soluble 5-hydroxymethylfurfural (HMF) was formed by condensation of hydroxaldehyde between glucose molecules, which is considered to be an important unit of the next step of the reaction. HMF is condensed by dehydration between molecules to form soluble polymers, then the aromatization reaction took place in the dehydrogenation of polymers. The aromatization products formed aromatic clusters through intermolecular dehydration [22,29,30,31,32,33,34,35,36,37]. The resulting aromatic compounds are thought to be the core of carbon nanospheres. When the aromatic group in the solution reaches the critical value, explosive nucleation will occur and the resulting carbon nucleus grows in the aqueous solution isotropic through diffusion. According to the principle of similarity, hydrophilic groups (such as hydroxyl groups, carboxyl groups) and hydrophobic substances (such as aromatic rings and ethers) are covalently bonded to form carbon nanospheres [35,38,39,40].

### 3.2. Characterization of CCS

**Figure 2:**
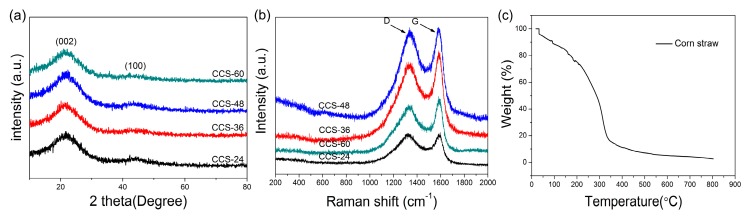
(**a**) XRD patterns and (**b**) Raman spectrum of CCS-24, CCS-36, CCS-48 and CCS-60 samples; (**c**) TG curve of corn straw.

Figure 2 shows the X-ray diffraction pattern of CCS-24, CCS-36, CCS-48 and CCS-60 samples. Two peaks can be observed near 22° and 44°, corresponding to the crystal plane (002) and (100) of typical disordered carbon. The intense peak (002) is commonly associated to the carbon interlayer-stacking structure and (100) to the interlayer reflection of the hexagonal carbon structure [41]. With the increase of reaction time in the HTC stage, the peak strength did not change obviously, which indicates that the reaction time has no obvious influence on the formation of amorphous carbon. Figure 2b displays the Raman spectrum of CCS-24, CCS-36, CCS-48 and CCS-60 samples. Although reaction time had no obvious influence on amorphous carbon, it changed the disorder degree of samples. The two characteristic peaks at 1340 and 1590 cm^−1^ correspond to the D and G bands. The D band is caused by the defects and disorder in the carbon material, and the G band is caused by the stretching of the C–C bond in the graphite. The I_D_/I_G_ ratio of the CCS-36 sample was 0.87, significantly lower than that of CCS-24 (I_D_/I_G_ = 0.92), CCS-48 (I_D_/I_G_ = 0.96) and CCS-60 (I_D_/I_G_ = 0.90), indicating the reduced disorder degree of the materials due to uniform distribution of carbon nanospheres. Figure 2c shows a thermogravimetric curve of corn straw. The first weight loss at less than 300 °C can be related to the presence of unbounded/physisorbed water. When the temperature exceeds 300 °C, the loss of weight is due to thermal degradation. The residual weight at 800 °C is estimated to be 2.63 wt.%.

**Figure 3:**
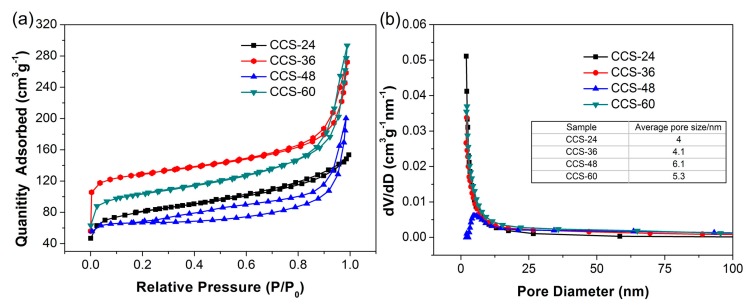
(**a**) Nitrogen adsorption–desorption isotherms and (**b**) pore sizes distribution of CCS-24, CCS-36, CCS-48 and CCS-60 samples.

In order to characterize the structure of carbon materials, the specific surface area and pore size distribution of samples are also measured through N_2_. BJH adsorption–desorption. As depicted in Figure 3a, the isotherm of CCS samples belongs to the type Ⅳ adsorption isotherm. At lower relative pressure, the curve rises upward, indicating the existence of micropores. These micropores are produced during the carbonization and activation stage. When the relative pressure is about 0.3, capillary condensation occurs, and the desorption isotherm lags behind the adsorption isotherm. The phenomenon of adsorption hysteresis that occur in CCS samples demonstrate the existence of mesopores (2–50 nm). When the relative pressure is close to 1, the adsorption of the macropores increases and the adsorption curve rises quickly. The specific surface areas of CCS-24, CCS-36, CCS-48 and CCS-60 are 183, 402, 201 and 339 m^2^ g^−1^, respectively. CCS-36 exhibits a larger specific surface area due to a larger number of spheres and cross-linked structure, which is more favorable for the intercalation and removal of lithium ions. Furthermore, the BJH (Barrett-Joyner-Halenda) pore size distribution diagrams are shown in Figure 3b. The average pore sizes of CCS-24, CCS-36, CCS-48, CCS-60 are 4.0, 4.1, 6.1 and 5.3 nm, respectively. The pore size of the four samples is mainly between 2–10 nm, the existence of these mesopores can promote the infiltration of the materials in the electrolyte and facilitate the diffusion of lithium ions in the electrode materials. The total pore volumes of CCS-24, CCS-36, CCS-48, CCS-60 are 0.20, 0.27, 0.22 and 0.35 cm^3^ g^−1^, respectively. Comparing the specific surface area and total pore volume of CCS-48 and CCS-60, it is possible that with the increase of reaction time, some carbon nanospheres grow up and finally break down.

**Figure 4:**
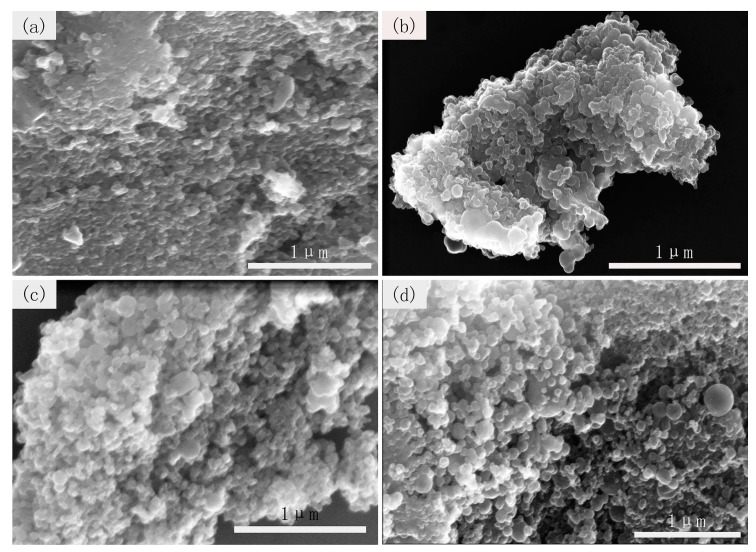
SEM images of (**a**) CCS-24, (**b**) CCS-36, (**c**) CCS-48, and (**d**) CCS-60 samples.

**Figure 5:**
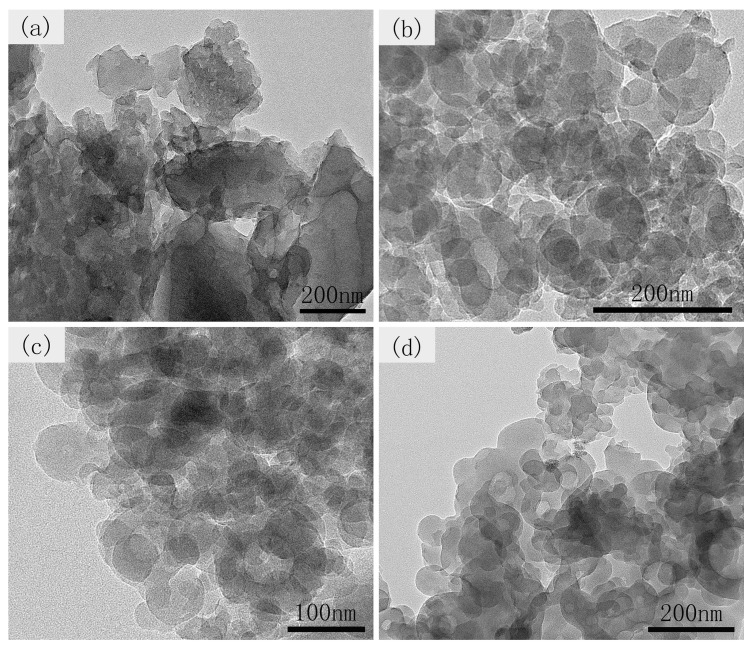
TEM images of (**a**) CCS-24, (**b**) CCS-36, (**c**) CCS-48, and (**d**) CCS-60 samples.

SEM and TEM are further used to characterize the morphology of materials. As presented in Figure 4a, the microstructure of CCS-24 sample is a large carbon block and a few spheres on the surface corresponding to the beginning of the carbon nanospheres formation after 24 h. Figure 4b shows a large number of carbon nanospheres with relatively uniform size and cross-linked structure, which is generated at 36 h. The cross-linked structure can shorten the distance between the lithium ion and electron, and thus improve the transmission efficiency. When the reaction time continued to increase, a large number of irregular-shaped carbon nanospheres generated together, as shown in Figure 4c,d. The occurrence of agglomeration will reduce the specific surface area of carbon materials, leading to the decrease of capacity. The TEM diagrams of CCS-24, CCS-36, CCS-48, CCS-60 are displayed in Figure 5. After comparison, it is observed that the hollow structure gradually formed, which is obvious with the increased reaction time. The hollow structure can reduce the structural damage caused by the intercalation of lithium ions and the collapse of the structure during the transmission process, thereby improving the cyclical stability of LIBs. In general, the formation of CCS occurs in the hydrothermal stage after 24 h, and the obtained carbon nanospheres possess a cross-linked structure. When the number of carbon nanospheres reached a certain level, the agglomeration appeared and became more and more serious over time.

### 3.3. Electrochemical Properties of CCS

**Figure 6:**
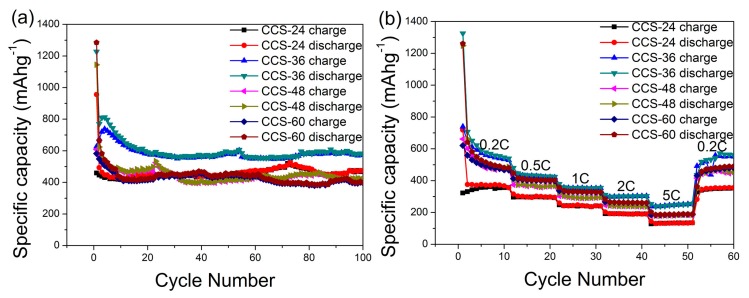
(**a**) Cycling performance of CCS samples at 0.2C and (**b**) rate performance of CCS samples.

The electrochemical cycling performance of CCS-24, CCS-36, CCS-48 and CCS-60 at 0.2C are compared in Figure 6a. After 100 cycles, the specific discharge capacities of samples are stable at 489, 577, 450 and 386 mA h g^−1^, respectively. After 100 cycles, the specific charge–discharge capacity increased from 24 h to 36 h. The specific charge–discharge capacity of CCS-36 remained at the highest level due to the favorable distribution and uniform size of carbon nanospheres, showing excellent battery-specific capacity and stable cycling performance. The specific charge and discharge capacity of CCS-48 and CCS-60 decreased gradually. Although the specific surface area and total pore volume of CCS-60 are larger than that of CCS-48, the pore size of CCS-60 is smaller than that of CCS-48, indicating that the size of the pore may have a greater effect on the capacity with a considerable increase in time. The charge–discharge curves of CCS samples do not coincide completely at the beginning of the first 10 cycles, while after 10 cycles, the charge–discharge curves basically coincide. This is due to the fact that lithium ions are used to form a solid electrolyte interfacial film (SEI) in the early charge–discharge stage, which causes the insertion and extraction of lithium ions in an unbalanced state. When a stable SEI film is formed, the insertion and extraction of lithium ions reach equilibrium state. To further evaluate the electrochemical performance of samples, the rate performance is also tested under various current densities (Figure 6b). After charge and discharge at the rate of 0.2, 0.5, 1, 2 and 5C, the specific capacities of the batteries almost returns to the initial one for the 0.2C rate. The unique spherical and cross-linked structures have shortened the transport distance between lithium ions and electrons and reduced the structural damage caused by the intercalation of lithium ions, leading to an excellent rate performance of anode materials.

**Figure 7:**
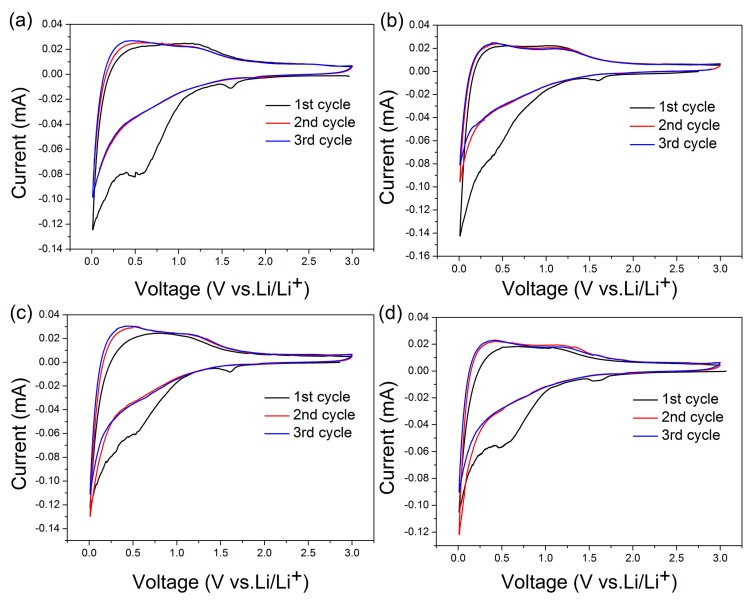
Cyclic Voltammograms of (**a**) CCS-24, (**b**) CCS-36, (**c**) CCS-48 and (**d**) CCS-60 samples.

Figure 7 displays the cyclic voltammogram curve of CCS-24, CCS-36, CCS-48, CCS-60 samples. The curves of the 2^nd^ and 3^rd^ laps basically coincide, which reflects the stability of electrochemical properties of CCS samples. During the first cycle of the CCS-36 sample, three reduction peaks appear near 0.2, 0.5 and 1.6 V. The peak near 0.2 V is formed due to lithium ion being embedded into the electrode material. The reduction peak near 0.5 V appears to be attributed to the catalytic reduction of electrolyte components on the surface of the active electrode to form SEI film. The reduction peak that appears near 1.6 V is considered to be caused by the reaction of lithium ion with the oxygen-containing functional group on the surface. In addition, there are two oxidation peaks near 0.25 V and 1.0 V. The flat oxidation peak at 0.25 V is corresponding to the extraction of lithium ions from the electrode. The hump at 1.0 V is formed by the adsorption and desorption of lithium ion in the mesopores of the material.

**Figure 8:**
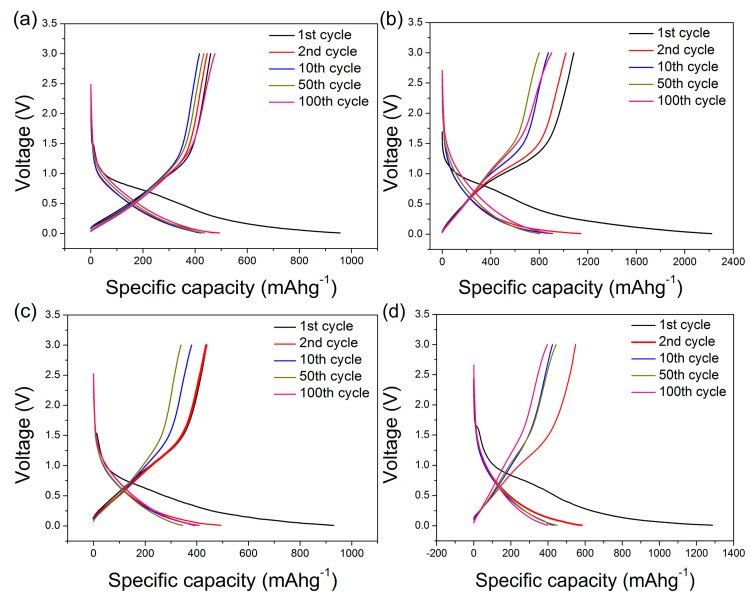
Charge-discharge curves of (**a**) CCS-24, (**b**) CCS-36, (**c**) CCS-48, and (**d**) CCS-60 samples.

**Figure 9:**
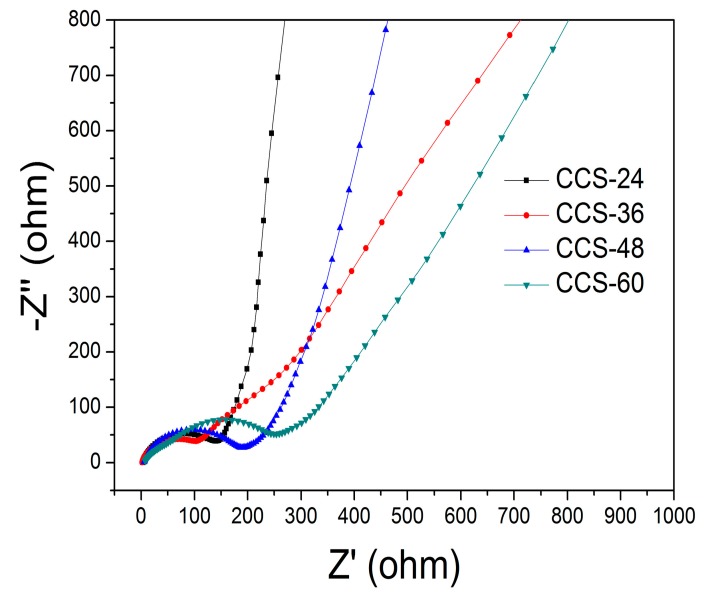
The impedance curves of CCS-24, CCS-36, CCS-48 and CCS-60 samples.

Figure 8 shows the charge–discharge curves of CCS-24, CCS-36, CCS-48, CCS-60 samples at 0.2C. The charge–discharge curve shows a weak discharge plateau near 0 V without another obvious charge–discharge plateau, which is a typical characteristic of the charge–discharge curve of carbon materials. In the first cycle, the specific discharge capacities of samples are 956, 1228, 1144 and 1285 mA h g^−1^, respectively. The coulombic efficiency of CCS-24, CCS-36, CCS-48 and CCS-60 are 48.1%, 51.0%, 53.1% and 45.4%, respectively. The coulombic efficiency of CCS samples is not particularly high, which is mainly determined by the characteristic of amorphous carbon. After the 100th cycle, the charge–discharge curves of the 2^nd^, 10^th^, 50^th^ and 100^th^ laps are basically coincidence, indicating that the unique spherical structure promotes the formation of stable SEI films to improve electrochemical stability. The impedance curve of samples CCS-24, CCS-36, CCS-48 and CCS-60 are shown in Figure 9. The semicircle diameter of CCS-36 is smaller than that of other samples, indicating that the charge transfer resistance is the smallest. This is due to the fact that the cross-linked structure and uniform distribution facilitate the transport of charge between the electrolyte and the interface of the material.

Table 1 shows biomass-derived carbons used as anodes in LIBs. From the comparison of different kinds of biomass as the electrode, it is clear that the structure of carbon materials and raw materials will lead to great differences in specific capacity. Table 2 presents the properties of different carbon nanospheres types. The structure and particle size of the carbon nanospheres have a great influence on the specific capacity. Although the experimental results of carbon materials prepared from corn straw have not been greatly improved due to the influence of the structure of raw materials, we still need to improve the experimental scheme and explore better preparation methods.

## 4. Conclusions

In summary, CCS is successfully synthesized through HTC from corn straw by carefully controlling the reaction time. Owing to the uniform distribution and cross-linked structure, CCS-36 has delivered excellent rate performance (577 mA h g^−1^ at 0.2C). When the current was restored to 0.2C again, the specific charge and discharge capacity quickly recovered to the initial value. Cellulose-derived carbon nanospheres provide more active sites for the intercalation of lithium ions by increasing the specific surface area, and thus, could further improve the specific capacity of LIBs. Meanwhile, the cross-linked structure not only shortens the distance between lithium ion and the electron, but also reduces the structural damage caused by the intercalation of lithium ions. In conclusion, we developed an environmental and efficient route to obtain cellulose-derived carbon nanospheres from corn straw, which not only realizes the secondary utilization of biomass waste, but also opens up a new opportunity for LIBs anodes.

## Figures and Tables

**Table 1:** Biomass-derived carbons used as anodes in LIBs.

Material	C Rate	Specific Capacity/ mA h g^−1^	Reference
Porous carbon from peanut shell	0.1	180	[37]
Carbon particle from coffee waste	0.27	285	[41]
Honeycomb carbon from sisal fiber	0.1	530	[42]
Carbon particle from apple waste	0.1	245	[43]
Carbon from cherry stones	5	200	[44]
Carbon sheet from banana fiber	0.1	310	[45]

**Table 2:** Performance comparison of different carbon nanospheres.

Carbon Nanospheres Type	Particle Size/nm	Specific Area/m^2^ g^−1^	C Rate	Specific Capacity/mA h g^−1^	Reference
Hollow nanospheres	50–150	369.44	0.2	489	[46]
Hollow microspheres	1000–3000	309.9	0.27	475	[47]
Hollow-in-hollow	350	1190.1	0.27	973	[48]
Nanoporous microspheres	700	2798.9	1.08	780	[49]
